# Hyperandrogenism secondary to topical testosterone exposure

**DOI:** 10.1186/1687-9856-2013-S1-P66

**Published:** 2013-10-03

**Authors:** A Carroll, S Moloney, NP Murphy

**Affiliations:** 1 Department of Endocrinology, Children’s University Hospital, Temple Street, Dublin 1, Ireland

## 

Topical testosterone gels are now a widely used method of testosterone replacement therapy and have been shown to be convenient and effective[1]. The unintentional transfer of testosterone gel to children or partners by skin contact is a rare but significant adverse effect[2].

A 3-year-old well girl was referred for assessment of precocious puberty. Pubic hair had been first noted by her mother 9 months earlier. Examination revealed a tall girl (>99.9^th^ centile) with no breast development but Tanner stage III pubic hair and cliteromegaly. Testosterone levels were elevated at 2.5nmol/l, as was her Androstenedione level at 1.1nmol/l. 17 OH P was normal and tumour markers were negative. Urine steroid profile was quantitatively normal but there was a modest increase in androgen metabolites. Bone age was advanced by 16 months. Ultrasound and MRI imaging of her ovaries and adrenals did not reveal a source of androgen production.

Upon further direct questioning, her father revealed he was using topical testosterone replacement therapy. Her father was advised of measures to reduce secondary exposure. Her repeat testosterone level fell to <0.7 nmol/l upon retesting 4 months later and the cliteromegaly resolved. At follow up a further six months later, testosterone level had risen to 2.5nmol/l. Switching to the use of IM preparations was encouraged. Follow-up testing after these measures were adopted revealed normal undetectable testosterone levels (<0.7nmol/l).

Once daily application of testosterone gels to the skin results in relatively stable and physiological testosterone levels in most users[3]. It is often favoured above the other methods of delivery of testosterone as it is painless, discrete and convenient to use. Even small quantities of transferred testosterone may result in clinical signs of hyperandrogenism.

When reviewing children with evidence of virilization, we must remember to question parents about the potential for exogenous androgen exposure.
